# Indomethacin Increases Quercetin Affinity for Human Serum Albumin: A Combined Experimental and Computational Study and Its Broader Implications

**DOI:** 10.3390/ijms21165740

**Published:** 2020-08-10

**Authors:** Hrvoje Rimac, Tana Tandarić, Robert Vianello, Mirza Bojić

**Affiliations:** 1 Department of Medicinal Chemistry, University of Zagreb Faculty of Pharmacy and Biochemistry, 10000 Zagreb, Croatia; mirza.bojic@pharma.unizg.hr; 2 Laboratory of Computational Modelling of Drugs, South Ural State University, 454008 Chelyabinsk, Russia; 3 Division of Organic Chemistry and Biochemistry, Ruđer Bošković Institute, 10000 Zagreb, Croatia; Tana.Tandaric@irb.hr (T.T.); Robert.Vianello@irb.hr (R.V.)

**Keywords:** human serum albumin, quercetin, indomethacin, pharmacokinetic interactions, fluorescence spectroscopy, docking, molecular dynamics, quantum chemistry

## Abstract

Human serum albumin (HSA) is the most abundant carrier protein in the human body. Competition for the same binding site between different ligands can lead to an increased active concentration or a faster elimination of one or both ligands. Indomethacin and quercetin both bind to the binding site located in the IIA subdomain. To determine the nature of the HSA-indomethacin-quercetin interactions, spectrofluorometric, docking, molecular dynamics studies, and quantum chemical calculations were performed. The results show that the indomethacin and quercetin binding sites do not overlap. Moreover, the presence of quercetin does not influence the binding constant and position of indomethacin in the pocket. However, binding of quercetin is much more favorable in the presence of indomethacin, with its position and interactions with HSA significantly changed. These results provide a new insight into drug-drug interactions, which can be important in situations when displacement from HSA or other proteins is undesirable or even desirable. This principle could also be used to deliberately prolong or shorten the xenobiotics’ half-life in the body, depending on the desired outcomes.

## 1. Introduction

Human serum albumin (HSA) is the most abundant protein in human plasma (60%, *w*/*w*) [1]. It is a 585-residue monomeric protein comprised of three homologous domains (I–III), each of which is composed of two subdomains (A and B) [2]. Its primary role in the body is preserving the oncotic pressure, but it also has additional functions, such as plasma pH regulation and transfer and storage of small hydrophobic molecules [3]. Two binding sites, where a majority of the ligands bind, are located in subdomains IIA and IIIA, and are also called warfarin and benzodiazepine sites, respectively [4]. The binding site located in the subdomain IIA appears to be the most prominent one and can accommodate structurally different ligands [5,6].

Various exogenous compounds bind to the IIA HSA subdomain, including drugs (e.g., indomethacin [5]), as well as flavonoids [7,8]. Flavonoids are a group of phenolic compounds, highly abundant in fruits and vegetables [9,10]. Apart from their antioxidative properties, which are the best described [11], they also have a significant role in preventing cardiovascular diseases [12,13,14]. Binding of flavonoids to HSA has been extensively studied using various techniques, such as fluorescence spectroscopy [7,8,15,16,17], circular dichroism [17,18,19,20], and molecular modeling [17,18,19,20,21], usually combining more than one approach. Since many different compounds bind in the IIA subdomain of HSA, there exists a possibility of their mutual competition for the binding sites and thus the potential displacement of either one or both ligands [22], in which the flavonoids can also participate [23]. However, there are several prerequisites that need to be met (e.g., kidney or liver diseases) for this type of interaction to have clinically important consequences [24,25,26]. On the other hand, to the best of our knowledge, a situation where one ligand increases the binding affinity of another ligand for HSA has not yet been reported in the literature, but could have important and even useful consequences. One such example is presented in this research.

**Figure 1:**
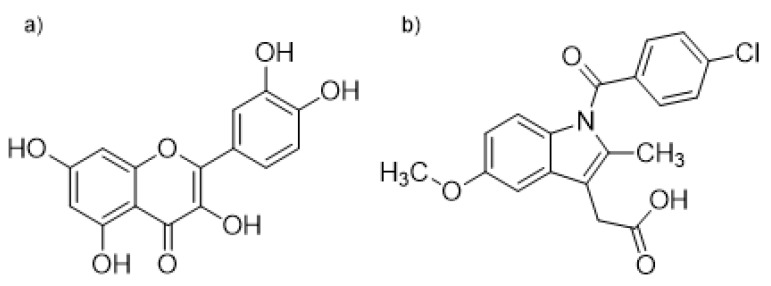
Structural formulas of (**a**) quercetin (QUE) and (**b**) indomethacin (IND).

We have investigated the interaction nature of indomethacin (IND), a well-known anti-inflammatory drug, and quercetin (QUE), one of the most abundant flavonoids in human nutrition, in binding to the binding site located in the IIA HSA domain. For this, we have combined experimental fluorometric experiments with docking, molecular dynamics simulations, and quantum chemical calculations. Binding of ligands in the IIA subdomain can easily be monitored by measuring the fluorescence intensity of the Trp214 residue and from the observed change in fluorescence their binding constants also can be calculated [27]. An alternative way to measure the binding of QUE to HSA is based on the fact that, when it is unbound in solution, QUE fluorescence is negligible, but when it is bound to HSA, its fluorescence increases dramatically. In this way both the binding of QUE to HSA and its dissociation from HSA caused by displacement can be observed [7,28,29]. The structures of QUE and IND are shown in Figure 1.

## 2. Results and Discussion

### 2.1. Simultaneous Binding of Indomethacin and Quercetin to HSA

For determination of the IND and QUE binding constants, Formulae 1–3 were used, as is explained in the Experimental section. Both IND and QUE bind in the vicinity of the Trp214 residue in the subdomain IIA [5,29], and their binding constants were determined to be (1.73 ± 0.64) × 10^5^ and (1.44 ± 0.05) × 10^5^ mol^−1^ dm^3^, respectively. To evaluate the risk of drug-drug interactions while binding to HSA, in vitro techniques have been used for a long time [22,30,31]. Results obtained in this way cannot be directly extrapolated to physiological conditions but can provide valuable insights [32]. In this interaction study, QUE fluorescence was used. QUE has a 5-OH group, which is known to quench its intrinsic fluorescence [28]. However, when it is bound to HSA, quercetin fluoresces at excitation and emission wavelengths of 450 and 500–540 nm, respectively. If quercetin is unable to bind to HSA or its binding affinity is changed due to an external factor, this can be observed from the intensity of their maximum fluorescence (*F*_max_) and the concentration needed to achieve it (*c*_max_). Using the obtained IND binding constant and Formulae 4–5, it is possible to determine the IND concentration needed to saturate a certain fraction of the HSA binding sites, with a presumed 1:1 binding ratio.

**Figure 2:**
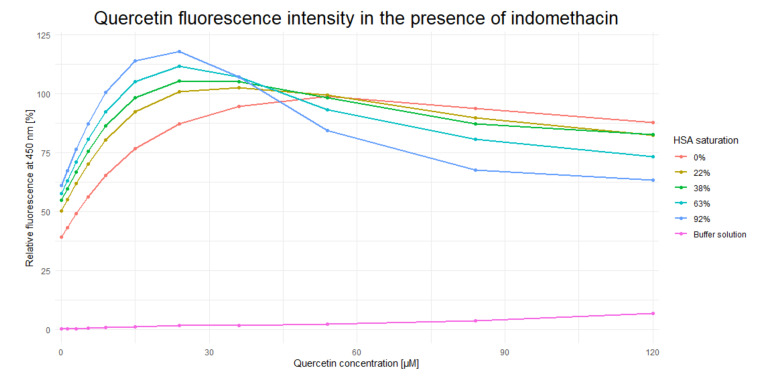
Dependence of quercetin fluorescence (*λ*_em_ = 526 nm) on indomethacin concentration.

After incubation with different concentrations of IND, QUE concentration was gradually increased until *F*_max_ was achieved (Figure 2). The presence of IND significantly increased the QUE’s *F*_max_ (by approximately 20%) and decreased its *c*_max_ from approximately 55 µM to 25 µM (i.e., by approximately 65%). These results indicate that IND and QUE do not bind in the same location within the HSA IIA subdomain. Moreover, the presence of IND significantly increases QUE’s binding constant through formation of a ternary HSA-IND-QUE complex. Additionally, the decrease in fluorescence intensity after reaching *F*_max_ can be explained by the inner filter effect, which plays a significant role in the ligand concentrations used in these experiments [33]. Alongside an increasing quercetin concentration, the indomethacin concentration needed to saturate the HSA follows a typical saturation curve: the higher the HSA saturation already is, a higher increase in indomethacin concentration is needed to change the saturation for a given percentage (for an HSA saturation of 22%, 38%, 63%, and 92% the corresponding indomethacin concentrations are 2.95, 5.82, 13.63, and 72.02 µM). This inner filter effect is clearly visible at a quercetin concentration of 120 µM, where its fluorescence decreases with an increase in indomethacin concentration. Additionally, at these high concentrations, contact quenching can also contribute to a fluorescence decrease, but at lower concentrations its effect is less pronounced. The obtained results are in accordance with results obtained by Zsila et al. [18] and Dufour and Dangles [7], as well as with the crystallographic structures obtained by Petitpas et al. [34] and Yamasaki et al. [35,36], stating that the binding site in the subdomain IIA of HSA is large enough to accommodate multiple ligands at the same time. This was also confirmed for the case of interactions between warfarin and quercetin [29]. The difference in QUE fluorescence is likely brought about either by (i) conformational changes within the ligand; (ii) potential changes in the size and shape of the IIA binding site caused by the presence of IND; or (iii) through the direct interaction of IND and QUE, which decreases QUE’s rotational and translational freedom and consequently stabilizes the ternary HSA-IND-QUE complex. In what follows, our computational analysis clearly shows that changes in the QUE fluorescence occur as a result of its modified interactions with the protein: in the presence of IND, QUE enters deeper into the HSA binding pocket and its binding energy is significantly enhanced, which likely restricts its conformational freedom, even at the expense of an unfavorable mutual interaction with IND.

### 2.2. Molecular Modeling of the Binary and Ternary HSA Complexes

**Figure 3:**
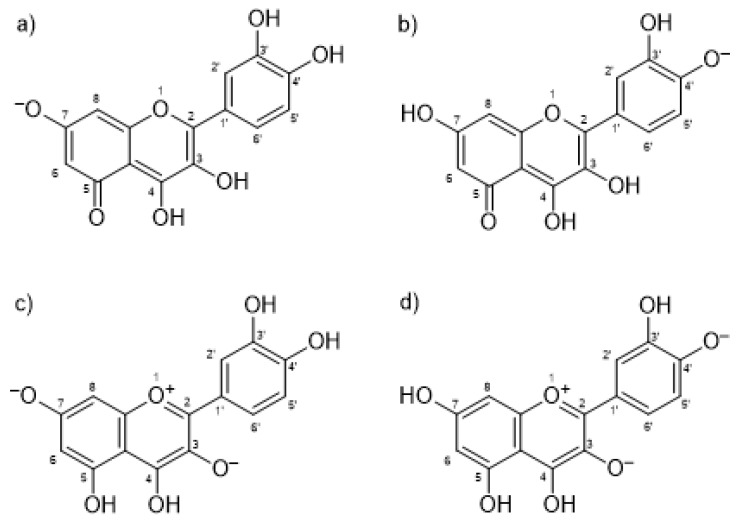
Docked quercetin species: (**a**) anion at Position 7; (**b**) anion at Position 4′; (**c**) fluorescent dianion at Positions 3 and 7; and (**d**) fluorescent dianion at Positions 3 and 4′.

To further investigate the IND-QUE interactions in binding to HSA, docking and molecular dynamics (MD) simulations were conducted. First, docking of quercetin to a crystallographic structure of HSA (PDB entry 2BXM) [5] was performed to see the difference in QUE binding in the presence and absence of IND, but also to obtain the initial quercetin and indomethacin poses for the MD simulations. Four different quercetin species were docked, two anions and two fluorescent dianion species (Figure 3), which were found to be most abundant according to Dangles et al. [37].

**Figure 4:**
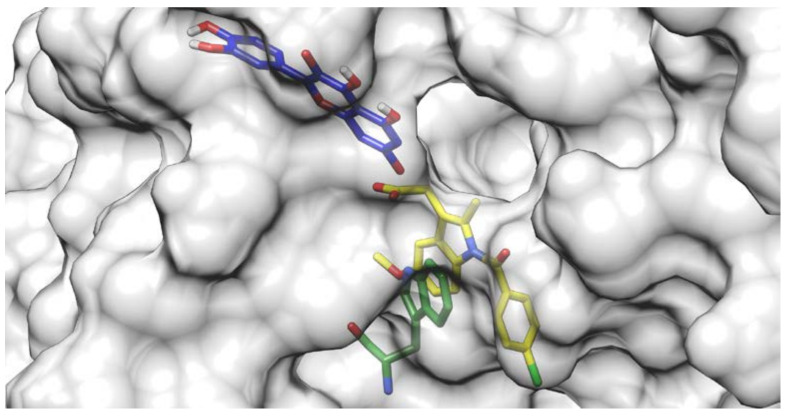
Quercetin anion at Position 7 (blue) docked to a crystallographic structure of indomethacin (yellow) bound to HSA. The Trp214 residue is depicted in green.

**Figure 5:**
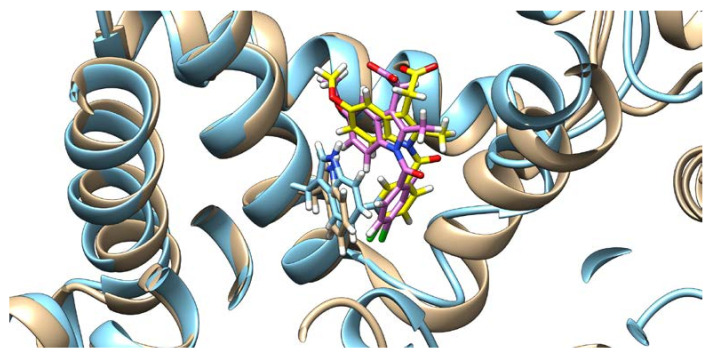
Indomethacin and HSA conformations before (indomethacin is depicted in pink and HSA in brown) and after (indomethacin is depicted in yellow and HSA in light blue) quercetin binding (quercetin is not shown), with the Trp214 shown.

Docking results of the QUE anion at Position 7 can be seen in Figure 4. QUE’s (blue) negatively charged 7-oxo group is located next to the IND’s (yellow) carboxylate moiety, suggesting an interaction between them. Since the results for all other quercetin species were virtually identical, the MD calculations were performed only for the quercetin anion at Position 7, as it is physiologically the most prevalent QUE protonation form at a pH of 7.4, with a determined p*K*a = 6.4 [38]. In addition, indomethacin was considered as a monoanionic system having a deprotonated carboxylic group, in line with its experimental p*K*a = 4.3 [39]. The MD simulations were conducted for both the binary complexes (HSA-IND and HSA-QUE), as well as for the ternary HSA-IND-QUE complex. The results show that there is no significant difference in the IND position between the binary HSA-IND complex (IND is depicted in pink and HSA in brown) and the ternary HSA-IND-QUE complex (IND is depicted in yellow and HSA in light blue) (Figure 5). The same can be said for HSA, with an exception of a slight loss of tertiary structure in one of the α helices.

**Figure 6:**
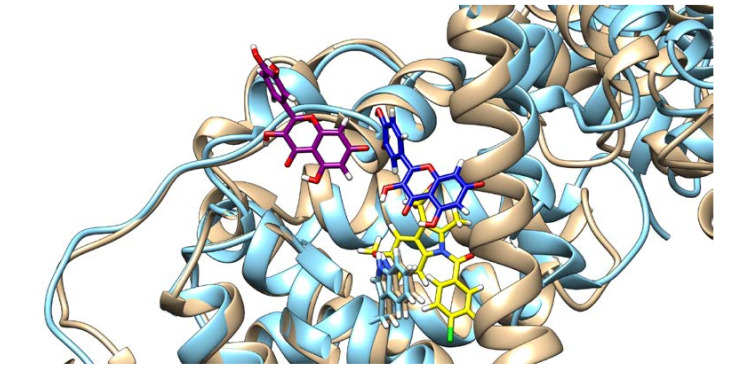
Quercetin poses and HSA conformation in the absence (quercetin is depicted in purple and HSA in brown) and in the presence of indomethacin (quercetin is depicted in blue, HSA in light blue, and indomethacin in yellow), with the Trp214 shown.

On the other hand, the presence of IND greatly influences QUE’s binding pose. As can be seen from Figure 6, in the absence of IND, QUE is located at the entrance of the IIA subdomain, but in its presence, QUE is positioned much deeper inside the hydrophobic pocket. This can explain QUE’s higher fluorescence intensity, as it is known that both the hydrophobic environment and the restriction of the intramolecular motion can cause an enhanced fluorescence intensity, as the energy cannot dissipate through other means [40,41].

**Figure 7:**
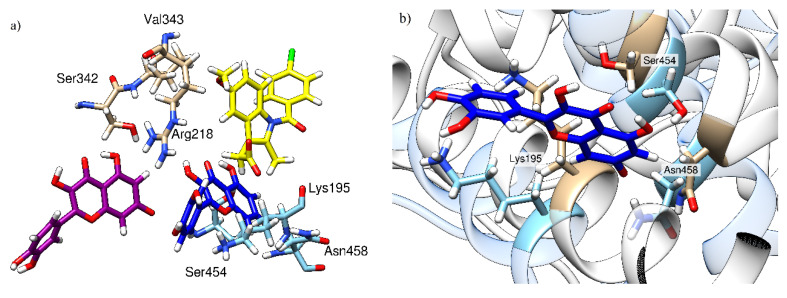
Relative quercetin orientation in the absence (purple) and in the presence (dark blue) of indomethacin (yellow) with three amino acids contributing the most to its binding. (**a**) Comparison of the relative quercetin locations in the absence and in the presence of indomethacin; (**b**) local changes in HSA conformation between a binary HSA-indomethacin complex (HSA depicted in brown, indomethacin is not shown) and a ternary HSA-indomethacin–quercetin complex (HSA is depicted in light blue, quercetin in dark blue, and indomethacin is not shown).

Molecular Mechanics Generalized Born Surface Area (MM-GBSA) analysis of the MD trajectories (Table 1) confirms that the presence of IND changes both the affinity of the HSA towards QUE and the position of the latter ligand within the binding site, which explains the observed changes in the fluorescence spectra reported earlier. Specifically, the calculated QUE’s binding free energy in the binary HSA-QUE complex is Δ*G*_bind_ = −20.0 kcal mol^−1^, which is increased to −30.4 kcal mol^−1^ in IND’s presence. We note that both of these Δ*G*_bind_ values are likely overestimated in absolute terms, which is a known limitation of the MM-GBSA approach, as extensively discussed in a recent review by Homeyer and Gohlke [42]. However, these authors also underlined its huge potential in predicting the relative binding energies in the biomolecular complexes. Therefore, in this context, these values evidently show that IND promotes the HSA-QUE interactions. With this in mind, our next task was to inspect how much of the mentioned 10.4 kcal mol^−1^ increase in the binding affinity can be attributed to the mutual interaction between QUE and IND themselves, and whether this represents the driving force for the change in the QUE position within the active site. To do so, we selected a representative structure of the ternary HSA-IND-QUE complex and extracted the geometry of the interacting ligands from the active site. These were kept frozen and submitted to DFT calculations at the M06–2X/6–31+G(d,p) level of theory, which gave a positive interaction free energy among the ligands of Δ*G*_int_ = +4.2 kcal mol^−1^. The latter indicates no favorable pairing between QUE and IND themselves in the ternary complex, leading to the conclusion that an increase in QUE binding in the presence of IND predominantly originates from the modified interactions with the protein. QUE’s orientation relative to the location of the indomethacin moiety and the most important amino acid residues contributing to quercetin binding are shown in Figure 7a. As can be seen, QUE’s relative position in the absence (purple) and in the presence (dark blue) of IND (yellow) significantly differs and the amino acids contributing the most to its binding are located at the opposite side of the binding pocket. In Figure 7b, the HSA conformational change can be observed, with an emphasis on the top three contributing residues (Lys195, Ser454, and Asn458). At this point it is important to delineate the two different effects exerted by the presence of IND: (1) it increases the HSA binding affinity for QUE; and (2) it results in a changed QUE fluorescence spectrum. Our analysis suggests that, while the former effect has no direct favorable contribution from the QUE-IND interactions themselves, the latter is brought about by a changed QUE environment in the ternary complex that has joint contributions from the vicinity of the IND and changed HSA residues.

Moreover, a decomposition of the calculated Δ*G*_bind_ values into contributions from individual residues clearly indicates different binding poses for QUE. Specifically, without IND, QUE binding is dominated by Ser342, Arg218, and Val343, whose contributions between −2.7 and −2.5 kcal mol^−1^ account for around 40% of the total binding free energy. Together with the remaining seven residues indicated in Table 1, they are responsible for around 60% of the binding. Interestingly, none of these 10 residues are governing QUE-HSA binding when IND is present, thus confirming a change in the binding position. The only exception is provided by Lys195, which increases its contribution from −0.60 to −1.88 kcal mol^−1^ in the presence of IND. This comes as a result of the fact that, without IND, Lys195 uses its side-chain protonated amino group (−NH_3_^+^) to form cation∙π interactions with the benzopyran-4-one fragment on QUE, while, when IND is present, these are replaced by a more favorable hydrogen bonding with the 3′- and 4′-hydroxyl groups on the phenyl ring, which results in an around three times increase in the Lys195 contribution. It is also interesting to observe that, without IND, QUE is positioned to strongly interact with Arg218 through favorable cation∙∙∙π interactions, which amount to over 10% of the total binding energy. Yet, when IND is present, this interaction is replaced by hydrogen bonding to the backbone amide of the same Arg218, but without a significant contribution to the binding free energy in that case (Table 1), thus again confirming a change in the binding pose.

Next, we note in passing that in the binary HSA-QUE complex, individual residue contributions are more equally distributed between the top contributors, whereas in the ternary HSA-IND-QUE complex, there is a large contribution of a single residue, Asn458, which exceeds 4 kcal mol^−1^. The latter is brought about by persistent hydrogen bonding between its amide N–H side chain and the anionic deprotonated phenoxide at Position 7 in QUE, with an average O(QUE)∙∙∙H–N(Asn458) value of 2.55 Å during the entire simulation. Nevertheless, despite this stable hydrogen bond, the overall number of hydrogen bonds that QUE establishes with all the binding site residues is decreased from 0.6 per ns when QUE is bound alone to 0.5 per ns when both ligands are present. This is consistent with the observed higher fluorescence of the ternary complex; QUE located deeper in the hydrophobic binding site is more rigid, which is accompanied by a slightly lower ability to create hydrogen bonds, which then results in the different spectroscopic responses demonstrated here.

Lastly, let us also mention that an analogous analysis of the IND binding (Table S1) shows that, on its own, IND is a much stronger HSA binder than QUE (Δ*G*_bind_ = −33.5 kcal mol^−1^), thus nicely reproducing a trend in the experimentally measured binding constants, and that this value is only moderately increased to −38.7 kcal mol^−1^ when QUE is present, thus tying in with the already presented conclusions. Decomposition of both of the mentioned binding energies into contributing residues reveals the repetition of many of them among the top contributors, therefore confirming similar binding poses for IND in both the secondary and ternary complexes. In addition, analysis of both Table 1 and Table S1 clearly indicates the significance of Trp214 in the binding of both ligands, which agrees with the experimental observations presented earlier.

Having all of these results at hand, the following conclusion can be made: IND is a stronger binder of the two, and it binds to HSA at its most favorable position, irrespective of the QUE presence. On the other hand, when alone, QUE binding is also favorable, but it is not maximized, as QUE binds in a shallower HSA region. The likely reason is that IND binding deepens the HSA binding site (by rearranging the conformations of residues in the vicinity of Lys195) (Figure 7b). This then allows QUE to enter deeper into the binding pocket and increase its binding affinity—a process QUE seems to be unable to undertake in the parent binding site—even at the expense of being unfavorably located in the close vicinity of IND. This kind of behavior changes in the secondary and tertiary HSA structure, as has already been observed for other ligands [43]. The next step in studying the HSA-IND-QUE interactions would be to perform so-called cluster-continuum calculations with individual amino acid residues (especially Lys195), to elucidate their individual contributions, as was done by Cappelli et al. [44] and Rybicka et al. [45].

## 3. Materials and Methods

Quercetin (purity ≥ 99%) and fatty acid-free HSA (purity ≥ 96%) were purchased from Sigma-Aldrich (St. Louis, MO, USA) and indomethacin (purity ≥ 99%) was purchased from Acros Organics, (Geel, Belgium). All chemicals were used without further purification. Binding of quercetin and indomethacin to HSA was studied using spectrofluorimetry, molecular docking, and molecular dynamics simulations.

### 3.1. Fluorescence Spectroscopy

HSA solutions were made daily before measurements in Dulbecco phosphate-buffered saline (PBS) (137 mM NaCl, 2.7 mM KCl, 8.1 mM Na_2_HPO_4_, 1.47 mM KH_2_PO_4_) [46]. Quercetin and indomethacin were dissolved in DMSO:PBS 1:1 and HSA was dissolved in PBS. In all experiments, the concentration of DMSO was held below 4%. This was done to prevent the conformational changes in HSA due to changes in the solvent dielectric constant. Steady-state fluorescence spectra were recorded using an OLIS RSM 1000F spectrofluorometer (Olis Inc., Bogart, GA, USA) equipped with a thermostated cell holder at 25 °C. Hellma Analytics 105.253-QS fluorescence cells with a light path of 10 × 2 mm (excitation × emission) were used. The excitation wavelength was 295 nm (tryptophan absorption maximum) and the emission spectra were recorded in the range 310–370 nm, where only HSA has fluorescent properties, with the observed maximum at 340 nm. All studies were performed in duplicate at 25 °C using 1.24 mm excitation and emission slit widths. All solutions were analyzed after 2 h incubation period.

For the binding constant determination, the HSA concentration was held constant at 1 µM while the ligand concentration varied between 0.03 and 10 µM and the effect of the DMSO as a co-solvent was annulated by adding a small aliquot of DMSO to a pure HSA solution. At the studied wavelength range both the HSA and quercetin absorb light. Based on the molar absorbance coefficients at 295 and 340 nm, and due to the relatively high concentrations, the inner filter effect cannot be neglected [47]. The observed fluorescence, *F*_obs_, was therefore corrected to *F*_corr_ according to Equation (1):

(1) $$F_{\mathrm{corr}}=F_{\mathrm{obs}}e^{\left(\frac{A_{\mathrm{ex}}+A_{\mathrm{em}}}{2}\right)}$$

where *A*_ex_ = *ε*_295_ × *c* × *l* is the absorbance at the excitation wavelength (*c* is ligand concentration and *l* = 1 cm) and *A*_em_ = *ε*_340_ × *c* × *l* is the absorbance at the emission wavelength (*l* = 0.2 cm).

Each spectrum obtained during titration is an average of 10,000 fluorescence spectra measured in 10 s. Equilibrium constants were calculated by a global fit at all wavelengths with SPECFIT [48,49,50,51] software. A single, significant, and spectrally active species was suggested by the singular value decomposition analysis and was attributed to the known spectrum of the HSA. This analysis also suggested a 1-to-1 complex formation and did not indicate any higher order complexes. Therefore, the proposed binding model is given by Equations (2) and (3), where *K* is the association constant of the complex:

(2) HSA+ligand⇄HSA−ligand

(3) $$K=\frac{\left[\mathrm{HSA}\;-\;\mathrm{ligand}\right]}{\left[\mathrm{HSA}\right]\left[\mathrm{ligand}\right]}$$

The association constant values calculated from fluorescence titrations for the suggested HSA–ligand complexes are given in mol^−1^ dm^3^. Higher values of *K* indicate stronger binding and higher complex stability.

For the HSA-IND-QUE interactions, the HSA concentration was kept at 6 µM while the fraction of HSA saturated by indomethacin varied: 0%, 22%, 38%, 63%, and 92%. The saturated fraction was calculated using Equations (4) and (5):

(4) $$K=\frac{\left[cL\right]}{[c_{f}]\left[L\right]}$$

(5) $$p=\frac{\left[cL\right]}{c}$$

where *K* is the binding constant, [*cL*] is the concentration of the HSA-ligand complex, [*c*_f_] is the concentration of the unbound HSA, [*L*] is the free ligand concentration, [*c*] is the total HSA concentration, and *p* is the fraction of saturated HSA. Flavonoid binding measurements were conducted at an excitation wavelength of 450 nm to maximize the fluorescence of the bound flavonoid, and the emission was recorded in the 466–580 nm range, with a maximum at 526 nm. Competition of indomethacin and quercetin for binding to HSA was determined based on the maximum intensity of fluorescence (*F*_max_) of quercetin and the concentration needed to achieve it (*c*_max_), as was done previously [29].

### 3.2. Docking Studies

Docking studies were performed using AutoDock 4.2.6. (The Scripps Research Institute, La Jolla, CA, USA) [52], which uses dispersion, hydrogen bonds, electrostatic, and desolvation components for the determination of the most probable complex conformation. This program was used to obtain the initial binding poses of quercetin to HSA in the presence and in the absence of the indomethacin molecule. 3D coordinates of the HSA–indomethacin complex were taken from the RCSB Protein Data Bank (entry 2BXM) [5] and missing side chains were added. Additionally, water molecules were omitted from the structure, hydrogen atoms were introduced where necessary, and all Lys and Arg side chains were protonated, while all Asp and Glu side chains were deprotonated. Both the amino and carboxyl ends were charged, His242 was protonated at the N_ε_ position, while all other His side chains were protonated at the N_δ_ position, based on visual inspection. This resulted in a total charge of the HSA molecule of −14. The initial 3D quercetin conformation was determined using HyperChem 8.0 (Hypercube, Inc., Gainesville, FL, USA), and its charge was set to the most abundant anionic and fluorescent anion species at pH 7.4, taken from Dangles et al. [37]. The quercetin partial charges were calculated according to Ionescu et al. [53]. A grid map of size 80 × 80 × 80 Å was generated with a 0.375 Å spacing centered on the coordinates of the side-chain nitrogen in Trp214 in Sudlow’s site I in subdomain IIA by the AutoGrid program [52], and then the Lamarckian genetic algorithm (LGA) [54] was applied. The receptor molecule was regarded as rigid while all ligand single bonds could rotate freely during the Monte Carlo simulated annealing procedure. Ligand flexible docking simulations were performed with 100 runs, a population size of 150, 2.5 × 10^7^ energy evaluations, 27,000 was the number of generations, a rate of gene mutation of 0.02, and a rate of crossover of 0.08. The root-mean-square-deviation (RMSD) of 2.0 Å was used as a criterion for cluster analysis of the docking results (in order to determine if the two docked conformations were similar enough to be included in the same cluster). Verification and modification of the crystallographic structure, as well as the results visualization, and interpretation were conducted using UCSF Chimera 1.10.1. (University of California, San Francisco, CA, USA) [55].

### 3.3. Molecular Dynamics Simulations and Quantum Chemical Calculations

The starting point of the molecular dynamics (MD) simulations were structures obtained by the docking procedure described above involving the QUE molecule bound to the crystallographic structure of the HSA-IND complex. To parameterize the QUE and IND molecules, geometry optimization and RESP charge calculations were performed using the Gaussian 16 program at the HF/6–31G(d) level to be consistent with the employed GAFF force field, while the protein was modeled using the AMBER ff14SB force field. In total, three HSA complexes were prepared (HSA-IND, HSA-QUE, and HSA-IND-QUE). Such complexes were then solvated in a truncated octahedral box of TIP3P water molecules spanning a 20 Å-thick buffer, neutralized by an equivalent of Na^+^ ions and submitted to geometry optimization in the AMBER16 program [56], employing periodic boundary conditions in all directions. Optimized systems were gradually heated from 0 to 300 K and equilibrated during 30 ps using NVT conditions, followed by productive and unconstrained MD simulations of 300 ns, employing a time step of 2 fs at a constant pressure (1 atm) and temperature (300 K), the latter held constant using a Langevin thermostat with a collision frequency of 1 ps^−1^. Bonds involving hydrogen atoms were constrained using the SHAKE algorithm [57], while the long-range electrostatic interactions were calculated employing the Particle Mesh Ewald method [58]. The nonbonded interactions were truncated at 10.0 Å. The binding free energies, Δ*G*_bind_, of QUE and IND within the IIA binding pocket of HSA were calculated using the established MM-GBSA protocol [59,60] available in AmberTools16 [56], and in line with our earlier reports [61,62]. MM-GBSA is a widely used method for binding free energy calculations from snapshots of the MD trajectory with an estimated standard error of 1–3 kcal mol^−1^ [59]. For that purpose, 1000 snapshots collected from the last 30 ns of the corresponding MD trajectories were utilized. The calculated MM-GBSA binding free energies were decomposed into a specific residue contribution on a per-residue basis according to the established procedure [63,64]. This protocol calculates contributions to Δ*G*_bind_ arising from each amino acid residue and identifies the nature of the energy change in terms of the interaction and solvation energies or entropic contributions. Lastly, in order to evaluate a mutual interaction between the QUE and IND themselves in the ternary HSA-IND-QUE complex, a frozen geometry of both ligands, extracted from the representative structure of the complex during MD simulations, was submitted to M06–2X/6–31+G(d,p) calculations. To account for the effect of the enzyme environment, we included the implicit CPCM solvent model, taking the dielectric constant of *ε* = 4 and all other parameters for pure water, as employed in many of our studies concerning various aspects of biomolecular systems [61,65,66]. The final interaction energy was calculated by comparing the so-obtained energy with energies of individual ligands, again frozen in the geometry they assume in the complex and considering only electronic energy contribution without thermal corrections.

## 4. Conclusions

Even though they both bind to the IIA subdomain of HSA, indomethacin and quercetin interact with different parts of the binding site. Additionally, binding of quercetin has little influence on the indomethacin affinity, while the presence of indomethacin significantly increases quercetin’s binding constant. This can be seen through the increase of quercetin’s fluorescence intensity (*F*_max_) and the decrease in its concentration needed to achieve it (*c*_max_). In the absence of indomethacin, quercetin binds at the entrance of the IIA binding site, but the presence of indomethacin changes the conformations of the amino acid residues inside the binding site. This allows quercetin to bind deeper into the binding pocket, without changing the indomethacin conformation. Even though this new quercetin conformation results in a stronger binding to HSA, quercetin is unable to perform this HSA conformational change on its own, due to its weaker binding constant compared to indomethacin.

Apart from the regular, competitive displacement interactions and non-interactions (e.g., warfarin-quercetin [29]), the obtained insight into the binding process suggests a third option for ligand-ligand interactions in a simultaneous binding to the same protein binding site, where one ligand increases the binding constant of the other ligand. At this moment, we are not aware of other studies that have reported similar results, which could have important consequences for the fate of xenobiotics inside the body. In most cases, pharmacokinetic interactions involving displacement of xenobiotics from their carrier proteins is undesirable as it can lead to their higher active concentrations and potential adverse effects, or faster elimination and reduced efficiency (e.g., in cases of antibiotics which have time-dependent effectiveness [6]). However, in certain cases, displacement of xenobiotics is desirable, as it reduces their toxicity (e.g., displacement of ochratoxin [67] or aflatoxin B_1_ [68] from HSA). In this regard, knowing to which one of the three possible types of ligand-ligand interactions our xenobiotic is subject to, can be of great importance in optimizing its desirable effects and preventing its undesirable effects, such as deliberately prolonging or shortening the xenobiotic half-life.

## Figures and Tables

**Table 1:** Contribution of the top individual residues to the binding of quercetin to HSA in the absence and in the presence of indomethacin obtained with the MM-GBSA approach. All values are in kcal mol^–1^ (# means number).

HSA-Quercetin Complex			HSA-Indomethacin-Quercetin Complex		
Residue Name	Residue #	Contribution to Δ*G*_bind_	Residue Name	Residue #	Contribution to Δ*G*_bind_
Ser	342	−2.70	Asn	458	−4.17
Arg	218	−2.69	Lys	195	−1.88
Val	343	−2.50	Ser	454	−1.85
Pro	447	−1.09	Val	455	−1.68
Trp	214	−0.63	Tyr	452	−1.59
Lys	195	−0.60	Ala	194	−1.25
Ser	192	−0.53	Leu	198	−0.80
Gln	196	−0.33	Asp	451	−0.77
Glu	450	−0.32	Ala	191	−0.48
Met	446	−0.23	Glu	188	−0.48
**Total Δ*G*_bind_ (Quercetin)**		**−20.0**	**Total Δ*G*_bind_ (Quercetin)**		**−30.4**

## Supplementary Materials

Supplementary materials can be found at https://www.mdpi.com/1422-0067/21/16/5740/s1. Table S1. Contribution of top individual residues to the binding of indomethacin to HSA in the absence and in presence of quercetin obtained with the MM-GBSA approach. All values are in kcal mol^−1^.

## Abbreviations

*c*_max_	Concentration Needed to Achieve Maximum Fluorescence Intensity
CPCM	Conductor-like Polarizable Continuum Model
*F*_max_	Maximum Fluorescence Intensity
HSA	Human Serum Albumin
LGA	Lamarckian Genetic Algorithm
MD	Molecular Dynamics
MM-GBSA	Molecular Mechanics Generalized Born Surface Area
IND	Indomethacin
QUE	Quercetin

## References

[1] Peters T. (1996). Metabolism: Albumin in the Body. All about Albumin.

[2] Carter D.C., He X.M. (1990). Structure of Human Serum Albumin. Science.

[3] Peters T. (1996). Ligand Binding by Albumin. All about Albumin.

[4] Sudlow G., Birkett D.J., Wade D.N. (1975). Characterization of Two Specific Drug Binding Sites on Human Serum Albumin. Mol. Pharmacol..

[5] Ghuman J., Zunszain P.A., Petitpas I., Bhattacharya A.A., Otagiri M., Curry S. (2005). Structural Basis of the Drug-Binding Specificity of Human Serum Albumin. J. Mol. Biol..

[6] Rimac H., Debeljak Ž., Bojić M., Miller L. (2017). Displacement of Drugs from Human Serum Albumin: From Molecular Interactions to Clinical Significance. Curr. Med. Chem..

[7] Dufour C., Dangles O. (2005). Flavonoid-Serum Albumin Complexation: Determination of Binding Constants and Binding Sites by Fluorescence Spectroscopy. Biochim. Biophys. Acta-Gen. Subj..

[8] Rimac H., Debeljak Ž., Šakić D., Weitner T., Gabričević M., Vrček V., Zorc B., Bojic M. (2016). Structural and Electronic Determinants of Flavonoid Binding to Human Serum Albumin: An Extensive Ligand-Based Study. RSC Adv..

[9] Pérez-Jiménez J., Fezeu L., Touvier M., Arnault N., Manach C., Hercberg S., Galan P., Scalbert A. (2011). Dietary Intake of 337 Polyphenols in French Adults. Am. J. Clin. Nutr..

[10] Mabry T.J., Markham K.R., Thomas M.B. (1970). The Systematic Identification of Flavonoids.

[11] Bors W., Michel C., Stettmaier K. (1997). Antioxidant Effects of Flavonoids. BioFactors.

[12] Dauchet L., Amouyel P., Hercberg S., Dallongeville J. (2006). Fruit and Vegetable Consumption and Risk of Coronary Heart Disease: A Meta-Analysis of Cohort Studies. J. Nutr..

[13] Bojić M., Debeljak Ž., Medić-Šarić M., Tomičić M. (2012). Interference of Selected Flavonoid Aglycons in Platelet Aggregation Assays. Clin. Chem. Lab. Med..

[14] Arts I.C.W., Hollman P.C.H. (2005). Polyphenols and Disease Risk in Epidemiologic Studies. Am. J. Clin. Nutr..

[15] Bi S., Ding L., Tian Y., Song D., Zhou X., Liu X., Zhang H. (2004). Investigation of the Interaction between Flavonoids and Human Serum Albumin. J. Mol. Struct..

[16] Khan M.K., Rakotomanomana N., Dufour C., Dangles O. (2011). Binding of Citrus Flavanones and Their Glucuronides and Chalcones to Human Serum Albumin. Food Funct..

[17] Dai J., Zou T., Wang L., Zhang Y., Liu Y. (2014). Investigation of the Interaction between Quercetin and Human Serum Albumin by Multiple Spectra, Electrochemical Impedance Spectra and Molecular Modeling. Luminescence.

[18] Zsila F., Bikádi Z., Simonyi M., Bika Z. (2003). Probing the Binding of the Flavonoid, Quercetin to Human Serum Albumin by Circular Dichroism, Electronic Absorption Spectroscopy and Molecular Modelling Methods. Biochem. Pharmacol..

[19] Mahesha H.G., Singh S.A., Srinivasan N., Appu Rao A.G. (2006). A Spectroscopic Study of the Interaction of Isoflavones with Human Serum Albumin. FEBS J..

[20] Jurasekova Z., Marconi G., Sanchez-Cortes S., Torreggiani A. (2009). Spectroscopic and Molecular Modeling Studies on the Binding of the Flavonoid Luteolin and Human Serum Albumin. Biopolymers.

[21] Feroz S.R., Mohamad S.B., Bakri Z.S.D., Malek S.N.A., Tayyab S. (2013). Probing the Interaction of a Therapeutic Flavonoid, Pinostrobin with Human Serum Albumin: Multiple Spectroscopic and Molecular Modeling Investigations. PLoS ONE.

[22] Kragh-Hansen U. (1981). Molecular Aspects of Ligand Binding to Serum Albumin. Pharmacol. Rev..

[23] Poór M., Boda G., Mohos V., Kuzma M., Bálint M., Hetényi C., Bencsik T. (2018). Pharmacokinetic Interaction of Diosmetin and Silibinin with Other Drugs: Inhibition of CYP2C9-Mediated Biotransformation and Displacement from Serum Albumin. Biomed. Pharmacother..

[24] DeVane C.L. (2002). Clinical Significance of Drug Binding, Protein Binding, and Binding Displacement Drug Interactions. Psychopharmacol. Bull..

[25] Hochman J., Tang C., Prueksaritanont T. (2015). Drug-Drug Interactions Related to Altered Absorption and Plasma Protein Binding: Theoretical and Regulatory Considerations, and an Industry Perspective. J. Pharm. Sci..

[26] Benet L.Z., Hoener B.-A. (2002). Changes in Plasma Protein Binding Have Little Clinical Relevance. Clin. Pharmacol. Ther..

[27] Eftink M.R., Ghiron C.A. (1981). Fluorescence Quenching Studies with Proteins. Anal. Biochem..

[28] Wolfbeis O.S., Begum M., Geiger H. (1984). Fluorescence Properties of Hydroxy- and Methoxyflavones and the Effect of Shift Reagents. Z. Naturforsch..

[29] Rimac H., Dufour C., Debeljak Ž., Zorc B., Bojić. M. (2017). Warfarin and Flavonoids Do Not Share the Same Binding Region in Binding to the IIA Subdomain of Human Serum Albumin. Molecules.

[30] D’Arcy P.F., McElnay J.C. (1982). Drug Interactions Involving the Displacement of Drugs from Plasma Protein and Tissue Binding Sites. Pharmacol. Ther..

[31] Sjöholm I., Ekman B., Kober A., Ljungstedt-Påhlman I., Seiving B., Sjödin T. (1979). Binding of Drugs to Human Serum Albumin: XI. The Specificity of Three Binding Sites as Studied with Albumin Immobilized in Microparticles. Mol. Pharmacol..

[32] Schmidt S., Gonzales D., Derendorf H. (2010). Significance of Protein Binding in Pharmacokinetics and Pharmacodynamics. J. Pharm. Sci..

[33] Credi A., Prodi L. (2014). Inner Filter Effects and Other Traps in Quantitative Spectrofluorimetric Measurements: Origins and Methods of Correction. J. Mol. Struct..

[34] Petitpas I., Bhattacharya A.A., Twine S., East M., Curry S. (2001). Crystal Structure Analysis of Warfarin Binding to Human Serum Albumin. Anatomy of Drug Site I. J. Biol. Chem..

[35] Yamasaki K., Maruyama T., Kragh-Hansen U., Otagiri M. (1996). Characterization of Site I on Human Serum Albumin: Concept about the Structure of a Drug Binding Site. Biochim. Biophys. Acta Protein Struct. Mol. Enzymol..

[36] Yamasaki K., Maruyama T., Takadate A., Suenaga A., Kragh-Hansen U., Otagiri M. (2004). Characterization of Site I of Human Serum Albumin Using Spectroscopic Analyses: Locational Relations between Regions Ib and Ic of Site I. J. Pharm. Sci..

[37] Dangles O., Dufour C., Bret S. (1999). Flavonol-Serum Albumin Complexation. Two-Electron Oxidation of Flavonols and Their Complexes with Serum Albumin. J. Chem. Soc. Perkin Trans..

[38] Álvarez-Diduk R., Ramírez-Silva M.T., Galano A., Merkoçi A. (2013). Deprotonation Mechanism and Acidity Constants in Aqueous Solution of Flavonols: A Combined Experimental and Theoretical Study. J. Phys. Chem. B.

[39] Zhou C., Jin Y., Kenseth J.R., Stella M., Wehmeyer K.R., Heineman W.R. (2005). Rapid pKa Estimation Using Vacuum-Assisted Multiplexed Capillary Electrophoresis (VAMCE) with Ultraviolet Detection. J. Pharm. Sci..

[40] Guharay J., Dennison S.M., Sengupta P.K. (1999). Influence of Different Environments on the Excited-State Proton Transfer and Dual Fluorescence of Fisetin. Spectrochim. Acta-Part A Mol. Biomol. Spectrosc..

[41] Sarkar M., Sengupta P.K. (1991). Influence of Different Micellar Environments on the Excited-State Proton-Transfer Luminescence of 3-Hydroxyflavone. Chem. Phys. Lett..

[42] Homeyer N., Gohlke H. (2012). Free Energy Calculations by the Molecular Mechanics Poisson-Boltzmann Surface Area Method. Mol. Inform..

[43] Balaei F., Ghobadi S. (2019). Hydrochlorothiazide Binding to Human Serum Albumin Induces Some Compactness in the Molecular Structure of the Protein: A Multi-Spectroscopic and Computational Study. J. Pharm. Biomed. Anal..

[44] Cappelli C., Bronco S., Monti S. (2005). Computational Study of Conformational and Chiroptical Properties of (2R,3S,4R)-(+)-3,3′,4,4′,7-Flavanpentol. Chirality.

[45] Rybicka A., Longhi G., Castiglioni E., Abbate S., Dzwolak W., Babenko V., Pecul M. (2016). Thioflavin T: Electronic Circular Dichroism and Circularly Polarized Luminescence Induced by Amyloid Fibrils. ChemPhysChem.

[46] Dulbecco R., Vogt M. (1954). Plaque Formation and Isolation of Pure Lines with Poliomyelitis Viruses. J. Exp. Med..

[47] Lakowicz J.R., Lakowicz J.R. (2006). Principles of Fluorescence Spectroscopy.

[48] Gampp H., Maeder M., Meyer C.J., Zuberbühler A.D. (1985). Calculation of Equilibrium Constants from Multiwavelength Spectroscopic Data-I: Mathematical Considerations. Talanta.

[49] Gampp H., Maeder M., Meyer C.J., Zuberbühler A.D. (1985). Calculation of Equilibrium Constants from Multiwavelength Spectroscopic Data-II: Specfit: Two User-Friendly Programs in Basic and Standard FORTRAN 77. Talanta.

[50] Gampp H., Maeder M., Meyer C.J., Zuberbühler A.D. (1985). Calculation of Equilibrium Constants from Multiwavelength Spectroscopic Data-III: Model-Free Analysis of Spectrophotometric and ESR Titrations. Talanta.

[51] Gampp H., Maeder M., Meyer C.J., Zuberbühler A.D. (1986). Calculation of Equilibrium Constants from Multiwavelength Spectroscopic Data-IV: Model-Free Least-Squares Refinement by Use of Evolving Factor Analysis. Talanta.

[52] Morris G.M., Huey R., Lindstrom W., Sanner M.F., Belew R.K., Goodsell D.S., Olson A.J. (2009). AutoDock4 and AutoDockTools4: Automated Docking with Selective Receptor Flexibility. J. Comput. Chem..

[53] Ionescu C.-M., Sehnal D., Falginella F.L., Pant P., Pravda L., Bouchal T., Svobodová Vařeková R., Geidl S., Koča J. (2015). AtomicChargeCalculator: Interactive Web-Based Calculation of Atomic Charges in Large Biomolecular Complexes and Drug-like Molecules. J. Cheminform..

[54] Huey R., Morris G.M., Olson A.J., Goodsell D.S. (2007). A Semiempirical Free Energy Force Field with Charge-Based Desolvation. J. Comput. Chem..

[55] Pettersen E.F., Goddard T.D., Huang C.C., Couch G.S., Greenblatt D.M., Meng E.C., Ferrin T.E. (2004). UCSF Chimera-A Visualization System for Exploratory Research and Analysis. J. Comput. Chem..

[56] Case D.A., Betz R.M., Cerutti D.S., Darden T.A., Duke R.E., Giese T.J., Gohlke H., Goetz A.W., Homeyer N., Izadi S. Amber 2016; University of California, San Francisco: San Francisco, CA, USA. http://ambermd.org/doc12/Amber16.pdf.

[57] Ryckaert J.P., Ciccotti G., Berendsen H.J.C. (1977). Numerical Integration of the Cartesian Equations of Motion of a System with Constraints: Molecular Dynamics of n-Alkanes. J. Comput. Phys..

[58] Darden T., York D., Pedersen L. (1993). Particle Mesh Ewald: An N·log(N) Method for Ewald Sums in Large Systems. J. Chem. Phys..

[59] Genheden S., Ryde U. (2015). The MM/PBSA and MM/GBSA Methods to Estimate Ligand-Binding Affinities. Expert Opin. Drug Discov..

[60] Hou T., Wang J., Li Y., Wang W. (2011). Assessing the Performance of the MM/PBSA and MM/GBSA Methods. 1. The Accuracy of Binding Free Energy Calculations Based on Molecular Dynamics Simulations. J. Chem. Inf. Model..

[61] Tandarić T., Vianello R. (2019). Computational Insight into the Mechanism of the Irreversible Inhibition of Monoamine Oxidase Enzymes by the Antiparkinsonian Propargylamine Inhibitors Rasagiline and Selegiline. ACS Chem. Neurosci..

[62] Perković I., Raić-Malić S., Fontinha D., Prudêncio M., Pessanha de Carvalho L., Held J., Tandarić T., Vianello R., Zorc B., Rajić Z. (2020). Harmicines—Harmine and Cinnamic Acid Hybrids as Novel Antiplasmodial Hits. Eur. J. Med. Chem..

[63] Gohlke H., Kiel C., Case D.A. (2003). Insights into Protein-Protein Binding by Binding Free Energy Calculation and Free Energy Decomposition for the Ras-Raf and Ras-RalGDS Complexes. J. Mol. Biol..

[64] Rastelli G., Del Rio A., Degliesposti G., Sgobba M. (2010). Fast and Accurate Predictions of Binding Free Energies Using MM-PBSA and MM-GBSA. J. Comput. Chem..

[65] Kržan M., Keuschler J., Mavri J., Vianello R. (2020). Relevance of Hydrogen Bonds for the Histamine H2 Receptor-Ligand Interactions: A Lesson from Deuteration. Biomolecules.

[66] Maršavelski A., Vianello R. (2017). What a Difference a Methyl Group Makes: The Selectivity of Monoamine Oxidase B Towards Histamine and N-Methylhistamine. Chem.-A Eur. J..

[67] Poór M., Kunsági-Máté S., Bencsik T., Petrik J., Vladimir-Knežević S., Koszegi T. (2012). Flavonoid Aglycones Can Compete with Ochratoxin A for Human Serum Albumin: A New Possible Mode of Action. Int. J. Biol. Macromol..

[68] Tan H., Chen L., Ma L., Liu S., Zhou H., Zhang Y., Guo T., Liu W., Dai H., Yu Y. (2019). Fluorescence Spectroscopic Investigation of Competitive Interactions between Quercetin and Aflatoxin B1 for Binding to Human Serum Albumin. Toxins.

