# Supplementary material for: Indomethacin Increases Quercetin Affinity for Human Serum Albumin: A Combined Experimental and Computational Study and Its Broader Implications
Source: Int J Mol Sci. 2020 Aug 10;21(16):5740. doi: 10.3390/ijms21165740 (PMC7460863; doi:10.3390/ijms21165740)
Supplement: Supplementary file 1 [file ijms-21-05740-s001.pdf]

Table S1. Contribution of the top individual residues to the binding of indomethacin to HSA in the absence and in presence of quercetin obtained with the MM–GBSA approach. All values are in kcal mol^{-1}.

**HSA–Indomethacin Complex**			**HSA–Indomethacin–Quercetin Complex**		
**Residue Name**	**Residue #**	**Contribution to ΔG_{bind}**	**Residue Name**	**Residue #**	**Contribution to ΔG_{bind}**
Arg	218	–7.46	Lys	199	–4.07
Trp	214	–4.57	Trp	214	–2.65
Lys	199	–2.76	Leu	198	–2.10
Phe	211	–1.79	Phe	211	–1.62
Lys	195	–1.48	Arg	218	–1.59
Ser	202	–1.31	Lys	195	–1.59
Ala	215	–1.01	His	242	–1.16
Leu	198	–0.99	Ser	202	–1.15
Leu	203	–0.87	Ala	215	–0.97
Ala	210	–0.72	Leu	481	–0.84
**Total ΔG_{bind} (Indomethacin)**		**–33.5**	**Total ΔG_{bind} (Indomethacin)**		**–38.7**
